# Association of Serum Ferritin Levels Before Start of Conditioning With Mortality After alloSCT – A Prospective, Non-interventional Study of the EBMT Transplant Complications Working Party

**DOI:** 10.3389/fimmu.2020.00586

**Published:** 2020-04-15

**Authors:** Olaf Penack, Christophe Peczynski, Steffie van der Werf, Jürgen Finke, Arnold Ganser, Helene Schoemans, Jiri Pavlu, Riitta Niittyvuopio, Wilfried Schroyens, Leylagül Kaynar, Igor W. Blau, Walter J. F. M. van der Velden, Jorge Sierra, Agostino Cortelezzi, Gerald Wulf, Pascal Turlure, Montserrat Rovira, Zubeydenur Ozkurt, Maria J. Pascual-Cascon, Maria C. Moreira, Johannes Clausen, Hildegard Greinix, Rafael F. Duarte, Grzegorz W. Basak

**Affiliations:** ^1^Department of Hematology, Oncology, and Tumor Immunology, Charité – Universitätsmedizin Berlin, Berlin, Germany; ^2^EBMT Statistical Unit, Paris, France; ^3^EBMT Data Office, Leiden, Netherlands; ^4^Department of Medicine I, Medical Center, Faculty of Medicine, University of Freiburg, Freiburg, Germany; ^5^Hannover Medical School, Hanover, Germany; ^6^Department of Hematology, University Hospital Leuven, KU Leuven, Leuven, Belgium; ^7^Imperial College London, London, United Kingdom; ^8^HUCH Comprehensive Cancer Center, Helsinki, Finland; ^9^Antwerp University Hospital, Antwerp, Belgium; ^10^Medical Faculty, Erciyes University, Kayseri, Turkey; ^11^Medical Centre, Radboud University, Nijmegen, Netherlands; ^12^Hospital de la Santa Creu I Sant Pau, Barcelona, Spain; ^13^Fondazione IRCCS Ca’ Granda, University of Milan, Milan, Italy; ^14^Department of Hematology and Medical Oncology, Universitätsklinikum Göttingen, Göttingen, Germany; ^15^CHRU Limoges, Limoges, France; ^16^Hospital Clinic, Barcelona, Spain; ^17^Faculty of Medicine, Gazi University, Ankara, Turkey; ^18^Hospital Regional de Málaga, Málaga, Spain; ^19^Instituto National do Cancer, Rio de Janeiro, Brazil; ^20^Elisabethinen Hospital, Linz, Austria; ^21^Department of Hematology and Oncology, Medical University of Graz, Graz, Austria; ^22^Hospital Universitario Puerta de Hierro, Madrid, Spain; ^23^Department of Hematology and Oncology, Medical University of Warsaw, Warsaw, Poland

**Keywords:** transplantation, stem cell, immunology, biomarker, iron metabolism, ferritin

## Abstract

Elevated serum ferritin levels occur due to iron overload or during inflammation and macrophage activation. A correlation of high serum ferritin levels with increased mortality after alloSCT has been suggested by several retrospective analyses as well as by two smaller prospective studies. This prospective multicentric study aimed to study the association of ferritin serum levels before start of conditioning with alloSCT outcome. Patients with acute leukemia, lymphoma or MDS receiving a matched sibling alloSCT for the first time were considered for inclusion, regardless of conditioning. A comparison of outcomes between patients with high and low ferritin level was performed using univariate analysis and multivariate analysis using cause-specific Cox model. Twenty centers reported data on 298 alloSCT recipients. The ferritin cut off point was determined at 1500 μg/l (median of measured ferritin levels). In alloSCT recipients with ferritin levels above cut off measured before the start of conditioning, overall survival (HR = 2.5, CI = 1.5–4.1, *p* = 0.0005) and progression-free survival (HR = 2.4, CI = 1.6–3.8, *p* < 0.0001) were inferior. Excess mortality in the high ferritin group was due to both higher relapse incidence (HR = 2.2, CI = 1.2–3.8, *p* = 0.007) and increased non-relapse mortality (NRM) (HR = 3.1, CI = 1.5–6.4, *p* = 0.002). NRM was driven by significantly higher infection-related mortality in the high ferritin group (HR = 3.9, CI = 1.6–9.7, *p* = 0.003). Acute and chronic GVHD incidence or severity were not associated to serum ferritin levels. We conclude that ferritin levels can serve as routine laboratory biomarker for mortality risk assessment before alloSCT.

## Introduction

Allogeneic stem cell transplantation (alloSCT) is a curative treatment option for patients suffering from hematological malignancies and some other diseases. High treatment-associated mortality is a major difficulty of this procedure and predicting mortality is a clinical challenge. The current standard for alloSCT risk assessment is the use of clinical scores, such as the European Society for Blood and Marrow Transplantation (EBMT)-score (1), the Hematopoietic Cell Transplantation-Comorbidity Index (HCT-CI) (2), the Dana-Farber Cancer Institute (DFCI)-score (3), and a combination of such scores (4).

The use of biomarkers to further improve alloSCT risk assessment is an attractive option (5). Mortality in the first months/years after alloSCT is mainly due to leukemia relapse, infections or graft-versus-host disease (GVHD). In all these clinical situations serum ferritin, an acute phase and iron binding protein, has been demonstrated to be elevated (6–13). Based on this background results, several retrospective studies and meta-analyses have suggested that serum ferritin may be of use as a biomarker during alloSCT (7, 14–18).

Based on these preliminary results and on the fact that ferritin is a routine laboratory parameter assessed in patients undergoing alloSCT, we saw a strong rationale for investigating serum ferritin as a biomarker. The Transplant Complications Working Party (TCWP) of the EBMT performed a prospective, multicenter and non-interventional study to test whether the ferritin level evaluated prior to the start of conditioning therapy is an independent risk factor for increased mortality after alloSCT.

## Materials and Methods

### Data Source, Study Design, and Data Collection

We asked EBMT centers performing more than 50 alloSCT per year if they were willing to participate in this prospective study. Twenty centers in ten countries agreed to participate. Data collection for the EBMT registry was approved by the European Society for Blood and Marrow Transplantation and by the IRB of Charité Universitätsmedizin Berlin as well as by IRBs of the participating centers. Data were prospectively collected between 8/2014 and 2/2018. Consecutive alloSCT recipients with acute leukemia, lymphoma or myelodysplastic syndrome (MDS) receiving a first matched sibling alloSCT from peripheral blood, regardless of conditioning, were eligible, provided they had signed an informed consent document that permitted sharing of clinical data according to national rules. Basic data on patient and disease characteristics as well as longer term follow up was taken from minimal essential data (MED-A) forms, which are submitted from all consecutive patients to the central EBMT registry. In addition, we designed registration and MED-B/C forms that were prospectively collected and specific to this study. Treatment teams completed specific forms (MED-B/C) forms at the time of registration and at day + 100 after alloSCT. The MED-B/C form contained detailed information on ferritin serum levels prior to alloSCT, patient characteristics, infectious- as well as non-infectious complications, GVHD staging, morbidity and mortality. Ferritin levels were determined at time of hospital admission for alloSCT directly before start of conditioning therapy.

## Endpoints and Statistical Analyses

Patient, disease, and transplant-related characteristics for the two cohorts (ferritin levels prior to alloSCT above median/ferritin levels below median) were compared by using χ2 statistics for categorical variables and the Mann-Whitney test for continuous variables. Primary endpoint was the incidence of acute GVHD. Acute GVHD was picked as a primary endpoint because of our previous observation on a correlation of maximum Ferritin levels after alloSCT with acute GVHD severity (12). Secondary endpoints were relapse incidence (RI), non-relapse mortality (NRM), overall survival (OS), progression free survival (PFS), and the incidence of chronic GVHD. PFS was defined as survival with no evidence of relapse or progression. RI was defined as the probability of having had a relapse during follow up time. Death without experiencing a relapse was a competing event. NRM was defined as death without evidence of relapse or progression. OS was defined as the time from alloSCT to death, regardless of the cause. To define acute GVHD during the consensus process, we used the criteria established by the MAGIC group (19–21). To define chronic GVHD, we used the NIH 2014 criteria (20–22). Cumulative incidence was used to estimate the endpoints of NRM, RI, acute, and chronic GVHD to accommodate for competing risks. To study acute and chronic GVHD, we considered relapse and death to be competing events. Probabilities of OS and PFS were calculated using the Kaplan–Meier method. Univariate analyses were done using the Gray test for cumulative incidence functions and the log rank test for OS and PFS. A Cox proportional hazards model was used for multivariate regression. All variables differing significantly between the 2 groups or factors associated with one outcome in univariate analysis were included in the Cox model. The following variables entered the multivariate models as possible confounders: age, sex mismatch between recipient and donor, diagnosis, disease status, Karnofsky score, number of CD34 cells given, intensity of conditioning (EBMT definition: myeloablative conditioning (MAC) was defined as TBI > 6 gray or oral busulfan > 8 mg/kg or intravenous busulfan > 6.4 mg/kg), type of GVHD prophylaxis, ATG use, time from diagnosis to transplant, year of transplant and CMV status. As the number of variables was too high regarding the number of events, a stepwise selection using Akaike information criterion (AIC) was run for all the confounding factors. The difference between the two cohorts was then assessed in the final selected model.

Results were expressed as the hazard ratio (HR) with the 95% confidence interval (95% CI). Proportional hazards assumptions were checked systematically for all proposed models using the Grambsch-Therneau residual-based test. All tests were 2-sided. The type I error rate was fixed at 0.05 for the determination of factors associated with time-to-event outcomes. Statistical analyses were performed in November 2018 with R 3.4.2 (R Core Team (2017). R: A language and environment for statistical computing. R Foundation for Statistical Computing, Vienna, Austria^1^).

## Results

### Patients and Transplant Characteristics

The entry criteria for analysis of OS were fulfilled in 298 patients. The main patients and transplant characteristics that were included in the analysis of OS are described in Table 1. Most parameters were balanced between the two cohorts. However, a higher percentage of sex mismatch transplants in the direction of female to male were observed in the group of patients with ferritin above cut off before alloSCT. The ferritin cut off point was determined at 1500 μg/l (median of measured ferritin levels).

**TABLE 1:** Population characteristics.

	Ferritin <=1500 μ G/L (*N* = 153)	Ferritin > 1500 μ G/L (*N* = 145)	*P*-value
Year of transplant median (range) [IQR]	2016 (2014–2018)[2015–2017]	2015 (2014–2018)[2015–2016]	0.6
Patient age (years) Median (range) [IQR]	52 (17.1–71.3) [38.1–60.2]	53.2 (19–70.9) [42.9–62.3]	0.3
Time from diagnosis to transplant (months) median (range) [IQR]	5 (1–71) [3–8]	4 (1–61) [3–6]	0.05
Number of CD34 + cells infused (E + 06) median (range) [IQR]	5.9 (0.9–10.7) [4.5–7.2]	5.5 (0.6–10.2) [4.2–6.7]	0.1
Sex mismatch			0.012
Female to male	27 (18%)	43 (30%)	
Other combination	123 (82%)	98 (70%)	
Diagnosis			0.058
Acute leukemia	93 (61%)	107 (74%)	
Lymphoma	19 (12%)	12 (8%)	
MDS	41 (27%)	26 (18%)	
Disease status			0.2
CR	94 (64%)	98 (70%)	
Not in CR	54 (36%)	42 (30%)	
ATG			0.5
No	77 (50%)	79 (54%)	
Yes	76 (50%)	66 (46%)	
Conditioning intensity			0.9
MAC/CHEMO	30 (20%)	32 (22%)	
MAC/TBI	26 (17%)	25 (17%)	
RIC	95 (63%)	87 (61%)	
Karnovsky Score			0.8
< = 80	23 (16%)	21 (15%)	
90–100	125 (85%)	122 (85%)	
Missing	5	2	
Disease risk index			0.8
Low	6 (4%)	4 (3%)	
Intermediate	86 (59%)	88 (65%)	
High	48 (33%)	39 (29%)	
Very high	5 (3%)	5 (4%)	
Missing	8	9	

### Endpoints

In the present study, the incidence of acute GVHD grades II–IV and grades III–IV in the whole population at 100 days was 25% and 11%, respectively. The incidence of chronic GVHD and severe chronic GVHD at last follow up was 25.8% and 15.1%, respectively. We observed no differences in incidence and severity of acute GVHD as well as chronic GVHD in between the two cohorts. As expected, chronic GVHD incidence was significantly lower in alloSCT recipients receiving anti-T-cell globulin as part of the conditioning regimen (ATG, HR = 0.25, CI = 0.13–0.5, *p* < 0.0001).

**FIGURE 1:**
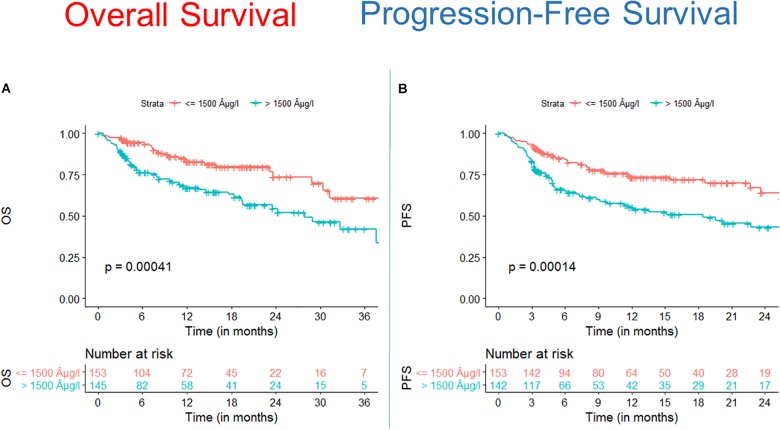
Survival after alloSCT in both cohorts according to ferritin serum levels prior to alloSCT. Overall survival (OS) **(A)** as well as progression free survival (PFS) **(B)** were significantly inferior in patients with high ferritin serum levels (blue line) as compared to patients with low ferritin levels (red line).

We found that OS and PFS of alloSCT recipients with ferritin levels above cut off measured before start of conditioning were significantly shorter as compared with the low ferritin cohort (Figure 1A, OS univariate HR = 2.3, CI = 1.4–3.6, *p* = 0.00041; multivariate HR = 2.5, CI = 1.5–4.1, *p* = 0.0005) (Figure 1B, PFS univariate HR = 2.1, CI = 1.4–3.2, *p* = 0.00014; multivariate HR = 2.4, CI = 1.6–3.8, *p* < 0.0001).

**FIGURE 2:**
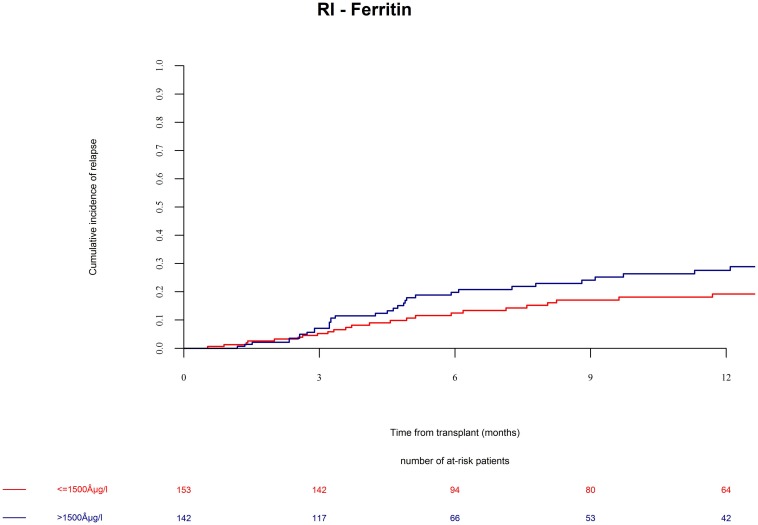
Relapse incidence (RI) after alloSCT in both cohorts according to ferritin serum levels prior to alloSCT. Relapse incidence was increased in patients with high ferritin serum levels (blue line) as compared to patients with low ferritin levels (red line).

Mortality was 29% at last follow up and with distribution of 15.0% relapse/progression as well as 14% NRM. Excess mortality in the high ferritin group was driven by higher relapse incidence as well as by higher NRM. We found that the incidence of relapse after alloSCT was 24.2% till the end of follow up. AlloSCT recipients with ferritin levels above cut off had significantly more relapses as compared with the low ferritin group (Figure 2, univariate HR = 1.7, CI = 1–2.8, *p* = 0.03; multivariate HR = 2.2, CI = 1.2–3.8, *p* = 0.007).

**FIGURE 3:**
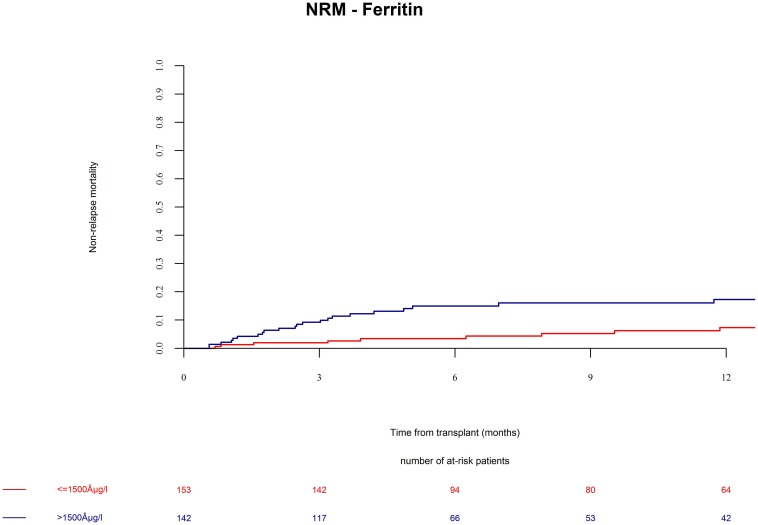
Non-relapse mortality (NRM) after alloSCT in both cohorts according to ferritin serum levels prior to alloSCT. NRM was increased in patients with high ferritin serum levels (blue line) as compared to patients with low ferritin levels (red line).

**FIGURE 4:**
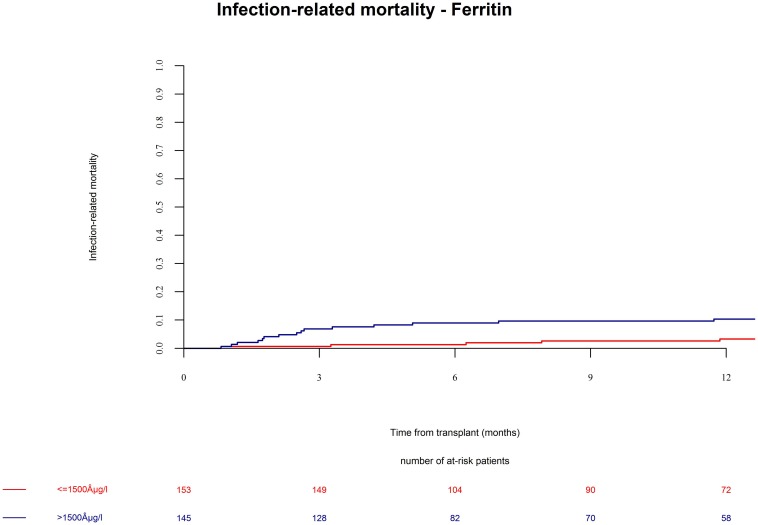
Infection-related mortality after alloSCT in both cohorts according to ferritin serum levels prior to alloSCT. Infection-related mortality was increased in patients with high ferritin serum levels (blue line) as compared to patients with low ferritin levels (red line).

Patients in the ferritin high group had significantly more NRM (Figure 3, univariate HR = 3.1, CI = 1.5–6.3, *p* = 0.002; multivariate HR = 3.1, CI = 1.5–6.4, *p* = 0.002). NRM was driven by significantly higher infection-related mortality in the high ferritin group (Figure 4, univariate HR = 3.9, CI = 1.6–9.7, *p* = 0.003; multivariate HR = 3.9, CI = 1.6–9.7, *p* = 0.003). A descriptive analysis of causes of death is given in Table 2.

**TABLE 2:** Mortality and cause of death in both cohorts.

Status at last follow up	Ferritin <= 1500 μ G/L (*N* = 153)	Ferritin > 1500 μ G/L (*N* = 145)
Alive	126 (82%)	92 (63%)
Dead	26 (18%)	53 (37%)
Death due to relapse or progression	15 (10%)	27 (19%)
Death without relapse	11 (7%)	26 (18%)
NRM infection related	5 (3%)	14 (10%)
NRM due to other causes	6 (4%)	12 (8%)

We conclude that serum ferritin levels prior to alloSCT are an independent risk factor for OS, relapse, and NRM at one year.

## Discussion

Our results from this prospective study indicate that serum ferritin prior to alloSCT is associated to mortality after alloSCT. This trial served as confirmation of previous retrospective studies, which delivered preliminary results pointing in the same direction (7, 14–18). Of note, the Center for International Blood and Marrow Transplantation Research (CIBMTR) published an evaluation on different biomarkers and found that ferritin levels above 2500μg/ml were not associated with inferior alloSCT outcome (23) contrasting previously published studies (7, 14–18) and our results.

A limitation of our clinical study is the lack of mechanistic insight on the role of ferritin in development of complications after alloSCT. In addition, our patient population was restricted to alloSCT from HLA-identical sibling donors. We are therefore unable to draw definite conclusions from these results regarding the association of ferritin levels with outcome in matched unrelated donor alloSCT or in haploidentical alloSCT, which are increasingly used.

An attractive feature of using ferritin as a biomarker for alloSCT outcome is that many severe clinical conditions occurring in the peri-transplant period are associated to high ferritin levels. First, high ferritin is related to iron overload although those factors are not always closely associated (10). Iron overload due to multiple pre-transplant red blood cell transfusion has been described to have negative impact on alloSCT outcome (7, 8, 11, 13). Second, ferritin levels are very high during severe graft-versus-host disease and during macrophage activation syndromes after alloSCT (12). Third, ferritin is an acute phase protein that is regularly elevated during acute and chronic infections (24, 25). Ferritin has been implicated in fungal growth and high ferritin levels have previously been suggested to be a risk factor for infections after alloSCT (26–28). In line with these data, our results indicated increased infection-related mortality in the group with higher ferritin levels. Fourth, ferritin levels have been associated with increased growth of acute myeloid leukemia (AML) (9, 16). So, taken together ferritin is elevated during tumor relapse and major infectious as well as non-infectious complications after alloSCT. Based on these considerations, ferritin might be a surrogate marker for organ dysfunction and it is understandable that it can serve as a biomarker for alloSCT mortality with significant prediction of tumor relapses as well as NRM.

In our study ferritin levels were not associated to a higher disease stage (CR/non-CR) or a longer time between diagnosis and alloSCT. In line with these findings, high ferritin levels were not associated to the diagnosis of MDS, where patients often receive multiple blood transfusions prior to alloSCT. There are two likely explanations: (1) Patients with multiple blood transfusions often receive drug treatment to reduce iron overload, and (2) The ferritin levels may not primarily reflect iron overload as discussed in the previous paragraph. Interestingly, a recent prospective study in patients with MDS and CMML undergoing alloSCT reported that administration of iron chelation therapy prior to HSCT was not associated to outcome. However, early iron reduction after alloSCT (started before d + 180) was associated to improved relapse free survival (29).

In conclusion, we found that high serum ferritin is an independent risk factor for increased mortality after alloSCT. The perspective of the future use of ferritin as biomarker will be likely a combination with clinical parameters such as the EBMT-score or the HCT-CI. However, to investigate such a combination or a combination with alternative biomarkers was not within the scope of our study and remains to be analyzed in the future.

## Data Availability Statement

The datasets generated for this study are available on request to the corresponding author.

## Ethics Statement

Ethical review and approval was not required for the study on human participants in accordance with the local legislation and institutional requirements. The patients/participants provided their written informed consent to participate in this study.

## Author Contributions

OP wrote the manuscript. CP analyzed the data. All authors read, edited, and approved the manuscript.

## Conflict of Interest

JF reports trials support and speakers honoraria by Medac, Neovii, and Riemser. The remaining authors declare that the research was conducted in the absence of any commercial or financial relationships that could be construed as a potential conflict of interest.

## Footnotes

1 https://www.R-project.org/

## References

[1] Gratwohl A Hermans J Goldman JM Arcese W Carreras E Devergie A Risk assessment for patients with chronic myeloid leukaemia before allogeneic blood or marrow transplantation. chronic leukemia working party of the european group for blood and marrow transplantation. *Lancet*. (1998) 352:1087–92. 9798583 10.1016/s0140-6736(98)03030-x

[2] Sorror ML Sandmaier BM Storer BE Maris MB Baron F Maloney DG Comorbidity and disease status based risk stratification of outcomes among patients with acute myeloid leukemia or myelodysplasia receiving allogeneic hematopoietic cell transplantation. *J Clin Oncol*. (2007) 25:4246–54. 17724349 10.1200/JCO.2006.09.7865

[3] Armand P Gibson CJ Cutler C Ho VT Koreth J Alyea EP risk index for patients undergoing allogeneic stem cell transplantation. *Blood*. (2012) 120:905–13. 10.1182/blood-2012-03-418202 22709687 PMC3412351

[4] Elsawy M Sorror ML. Up-to-date tools for risk assessment before allogeneic hematopoietic cell transplantation. *Bone Marrow Transplant*. (2016) 51:1283–300. 10.1038/bmt.2016.141 27272454

[5] Rowan CM Paczesny S. Biomarkers for early complications after hematopoietic stem cell transplantation. *Clin Lab Med*. (2019) 39:61–72. 10.1016/j.cll.2018.10.005 30709509 PMC7138508

[6] Armand P Kim HT Rhodes J Sainvil MM Cutler C Ho VT Iron overload in patients with acute leukemia or MDS undergoing myeloablative stem cell transplantation. *Biol Blood Marrow Transplant J Am Soc Blood Marrow Transplantat*. (2011) 17:852–60. 10.1016/j.bbmt.2010.09.006 PMC3954514 20854920

[7] Armand P Kim HT Virtanen JM Parkkola RK Itala-Remes MA Majhail NS Iron overload in allogeneic hematopoietic cell transplantation outcome: a meta-analysis. *Biol Blood Marrow Transplant J Am Soc Blood Marrow Transplant*. (2014) 20:1248–51. 10.1016/j.bbmt.2014.04.024 24769316 PMC4099413

[8] Armand P Sainvil MM Kim HT Rhodes J Cutler C Ho VT Does iron overload really matter in stem cell transplantation? *Am J Hematol*. (2012) 87:569–72. 10.1002/ajh.23188 22473510 PMC3358569

[9] Ihlow J Gross S Sick A Schneider T Florcken A Burmeister T AML: high serum ferritin at initial diagnosis has a negative impact on long-term survival. *Leuk Lymph*. (2019) 60:69–77. 10.1080/10428194.2018.1461860 29846127

[10] Jarisch A Salzmann-Manrique E Cario H Grosse R Soerensen J Fischer R Serum ferritin is not a reliable predictor to determine iron overload in thalassemia major patients post-hematopoietic stem cell transplantation. *Eur J Haematol*. (2018) 101:791–7. 30187571 10.1111/ejh.13169

[11] Leitch HA Fibach E Rachmilewitz E. Toxicity of iron overload and iron overload reduction in the setting of hematopoietic stem cell transplantation for hematologic malignancies. *Crit Rev Oncol Hematol*. (2017) 113:156–70. 10.1016/j.critrevonc.2017.03.002 28427505

[12] Nogai A Shi Y Perez-Hernandez D Cordes S Mengwasser J Mertlitz S Organ siderosis and hemophagocytosis during acute graft-versus-host disease. *Haematologica*. (2016) 101:e344–6. 27198715 10.3324/haematol.2016.144519 PMC4967586

[13] Wermke M Eckoldt J Gotze KS Klein SA Bug G de Wreede LC Theurl, and U. Platzbecker, Enhanced labile plasma iron and outcome in acute myeloid leukaemia and myelodysplastic syndrome after allogeneic haemopoietic cell transplantation (ALLIVE): a prospective, multicentre, observational trial. *Lancet Haematol*. (2018) 5:e201–10. 29628397 10.1016/S2352-3026(18)30036-X

[14] Chee L Tacey M Lim B Lim A Szer J Ritchie D. Pre-transplant ferritin, albumin and haemoglobin are predictive of survival outcome independent of disease risk index following allogeneic stem cell transplantation. *Bone Marrow Transplant*. (2017) 52:870–7. 10.1038/bmt.2017.51 28504664

[15] Laribi K Bolle D Alani M Ghnaya H Le Bourdelles S Besancon A de Materre, Prognostic impact of elevated pretreatment serum ferritin in patients with high-risk MDS treated with azacitidine. *Exp Hematol*. (2018) 65:34–7. 29883686 10.1016/j.exphem.2018.05.006

[16] Tachibana T Andou T Tanaka M Ito S Miyazaki T Ishii Y Clinical significance of serum ferritin at diagnosis in patients with acute myeloid leukemia: a YACHT multicenter retrospective study. *Clin Lymphom Myelo Leukem*. (2018) 18:415–21. 10.1016/j.clml.2018.03.009 29673622

[17] Tanaka M Kanamori H Matsumoto K Tachibana T Numata A Ohashi K Clinical significance of pretransplant serum ferritin on the outcome of allogeneic hematopoietic SCT: a prospective cohort study by the kanto study group for cell therapy. *Bone Marrow Transplant.* (2015) 50:727–33. 10.1038/bmt.2015.17 25730191

[18] Yan Z Chen X Wang H Chen Y Chen L Wu P Effect of pre-transplantation serum ferritin on outcomes in patients undergoing allogeneic hematopoietic stem cell transplantation: a meta-analysis. *Medicine*. (2018) 97:e10310. 10.1097/MD.0000000000010310 29979374 PMC6076067

[19] Harris AC Young R Devine S Hogan WJ Ayuk F Bunworasate U International, multicenter standardization of acute graft-versus-host disease clinical data collection: a report from the mount sinai acute GVHD international consortium. *Biol Blood Marrow Transplant*. (2016) 22:4–10. 10.1016/j.bbmt.2015.09.001 26386318 PMC4706482

[20] Schoemans HM Goris K Van Durm R Fieuws S De Geest S Pavletic SZ The eGVHD App has the potential to improve the accuracy of graft-versus-host disease assessment: a multicenter randomized controlled trial. *Haematologica*. (2018) 103:1698–707. 10.3324/haematol.2018.190777 29903762 PMC6165809

[21] Schoemans HM Lee SJ Ferrara JL Wolff D Levine JE Schultz KR position statement on standardized terminology & guidance for graft-versus-host disease assessment. *Bone Marrow Transplant*. (2018) 53:1401–15. 29872128 10.1038/s41409-018-0204-7 PMC6786777

[22] Jagasia MH Greinix HT Arora M Williams KM Wolff D Cowen EW National institutes of health consensus development project on criteria for clinical trials in chronic graft-versus-host disease: I. the 2014 diagnosis and staging working group report. *Biol Blood Marrow Transplant*. (2015) 21:389–401e1. 10.1016/j.bbmt.2014.12.001 25529383 PMC4329079

[23] Artz AS Logan B Zhu X Akpek G Bufarull RM Gupta V The prognostic value of serum C-reactive protein, ferritin, and albumin prior to allogeneic transplantation for acute myeloid leukemia and myelodysplastic syndromes. *Haematologica*. (2016) 101:1426–33. 27662010 10.3324/haematol.2016.145847 PMC5394859

[24] Chow JKL Ganz T Ruthazer R Simpson MA Pomfret EA Gordon FD Iron-related markers are associated with infection after liver transplantation. *Liver Transplant Off Pub Am Assoc Study Dis Int Liver Transplant Soc*. (2017) 23:1541–52. 10.1002/lt.24817 28703464 PMC5696081

[25] Miranda P Gil-Santana L Oliveira MG Mesquita ED Silva E Rauwerdink A Sustained elevated levels of C-reactive protein and ferritin in pulmonary tuberculosis patients remaining culture positive upon treatment initiation. *PLoS One* (2017) 12:e0175278. 10.1371/journal.pone.0175278 28384354 PMC5383283

[26] Almeida RS Brunke S Albrecht A Thewes S Laue M Edwards JE The hyphal-associated adhesin and invasin Als3 of Candida albicans mediates iron acquisition from host ferritin. *PLoS Pathog*. (2008) 4:e1000217. 10.1371/journal.ppat.1000217 19023418 PMC2581891

[27] Dadwal SS Tegtmeier B Liu X Frankel P Ito J Forman SJ Impact of pretransplant serum ferritin level on risk of invasive mold infection after allogeneic hematopoietic stem cell transplantation. *Eur J Haematol*. (2015) 94:235–42. 10.1111/ejh.12421 25082161

[28] Tachibana T Tanaka M Takasaki H Numata A Ito S Watanabe R Pretransplant serum ferritin is associated with bloodstream infections within 100 days of allogeneic stem cell transplantation for myeloid malignancies. *Int J Hematol*. (2011) 93:368–74. 10.1007/s12185-011-0784-0 21331523

[29] Cremers EMP de Witte T de Wreede L Eikema DJ Koster L van Biezen A A prospective non-interventional study on the impact of transfusion burden and related iron toxicity on outcome in myelodysplastic syndromes undergoing allogeneic hematopoietic cell transplantation(). *Leukem Lymph*. (2019) 60:2404–14. 10.1080/10428194.2019.1594215 30997844

[30] Penack O Peczynski C van der Werf S Mohty M Yakoub-Agha I Montoto S “Bone marrow transplantation association of serum ferritin levels before start of conditioning with mortality after alloSCT – a prospective, non-interventional study of the EBMT transplant complication working party,” in *Proceedings of the 45th Annual Meeting of the European-Society-for-Blood-and-Marrow-Transplantation (EBMT)*, Vol. 54 (Frankfurt: Springer Nature), 238–239.

